# Ten-Year Trends in the Morbidity of Diabetes Mellitus and Antidiabetic Drug Utilization in Croatia: A Study Based on Routinely Collected Data

**DOI:** 10.1155/2016/9837496

**Published:** 2016-07-04

**Authors:** Renata Pavlov, Ivančica Topličan, Mladenka Vrcić Keglević

**Affiliations:** ^1^Family Practice “Dr. Renata Pavlov”, Aleja lipa 2a, 10000 Zagreb, Croatia; ^2^Family Practice “Dr. Ivančica Topličan”, Aleja lipa 2a, 10000 Zagreb, Croatia; ^3^Foundation for the Development of Family Medicine in Croatia, Cresnjevec 32, 10000 Zagreb, Croatia

## Abstract

*Objectives*. To investigate trends of diabetes mellitus (DM) morbidity and antidiabetic drug utilization in Croatian primary health care (PHC) from 2005 to 2014.* Method.* Routinely collected morbidity data from all PHC units, presented in Croatian health-statistics yearbooks, were retrieved. Data on drug utilization were retrieved from the Annual Reports of the Croatian Agency for Medicinal Products and Medical Devices (ATC/DDD, antidiabetic, A10).* Results.* Total morbidity increased by 33.3% and DM increased by 65.6%, mostly in patients over age 65 (from 50% to 57%). Estimated DM prevalence in adults increased from 3.9% to 6.4%. Increased morbidity was followed by an even higher increase in drug utilization (120%). Metformin was first, with a constant increase (from 18% to 39%), followed by glimepiride, while glibenclamide use decreased. Total utilization of insulin increased even more, mostly for aspart (600%) and newly introduced glargine and detemir, while human insulin usage sharply decreased. Spending also increased, mostly for aspart (from 21% to 61% of total).* Conclusions*. Increased DM is followed by a higher increase in antidiabetic drug utilization; this trend will continue in the future. In Croatian PHC, metformin has primacy along with insulin analogues.

## 1. Introduction

Diabetes mellitus (DM) is a multifactorial disease that occurs in genetically susceptible individuals under the influence of environmental factors. Disorders in insulin secretion or insulin action, or both, lead to hyperglycaemia. In addition to the consequences of abnormal glucose metabolism, DM usually leads to long-term complications such as cardiovascular, ocular, neurological, and renal complications. According to World Health Organization (WHO) data, an estimated 1.5 million deaths were directly caused by diabetes in 2012, mainly because of those complications [1]. In Croatia, with 4.28 million inhabitants, DM complications were the sixth leading cause of death in women and the eighth in men older than 65 years in 2010 [2]. In 2010 and 2011, DM with its complications was the fourth highest-ranking reason for the hospitalization of elderly men and the third highest for elderly women in Croatia [3]. According to the Global Burden of Disease project data, the disability-adjusted life-years related to DM increased by 20% in Croatia during the period of 1990–2010 [4].

This chronic illness requires continuing medical care, including investments in health care personnel and facilities, as well as in pharmaceutical supplies. According to International Diabetes Federation, the majority of countries spend between 5% and 20% of their total health expenditure on diabetes. This is a significant challenge for healthcare systems and an obstacle to sustainable economic development [5]. In Croatia, around 10% of the health budget is spent on DM; of this, 85.7% is for the hospital treatment of DM complications, 8.8% is for antidiabetic drugs, 3.5% is for diabetologist care, and 2.04% is for primary care. 6.2% of total drug utilization in Croatia is spent on antidiabetics [6].

From the patient's perspective, DM is a lifelong, 24-hour-a-day condition. It is up to the person living with this condition to pay attention every day to eating, exercising, taking medication, testing glucose levels, and maintaining a normal body weight [7]. In addition to the deterioration in quality of life, indirect costs for patients and society are related to a decline in work productivity and losses due to absenteeism and early retirement [8, 9].

We did not find any published studies investigating the long-lasting morbidity trends of DM in Croatia, particularly concerning everyday practices and relating to the whole population. Therefore, this study was undertaken to determine the 10-year (2005–2014) morbidity trends of DM patients registered in primary health care (PHC) in Croatia and to determine the trends for antidiabetic drug utilization during that period of time.

## 2. Method and Materials

### 2.1. Study Design

This study was observational and retrospective, based on routinely collected data at the national level. We decided to use official national data because they were coming from everyday practices and they were related to the whole population. Every health care institution in Croatia is obliged to collect and refer to the Croatian National Institute of Public Health (CNIPH) the data on each patient visit to PHC, specialist-consultants, or in-hospital discharge. Furthermore, routinely collected data are shown to be a reliable source of information reflecting the everyday realities of health service delivery [10, 11]. In this study used morbidity data are related to the PHC level. PHC in Croatia is mostly based on family practice and paediatric services. Almost 100% of the population is registered with PHC; the adult population and school-age children are on family doctors' lists and the majority of pre-school-age children are on paediatricians' lists and the rest are on family doctors' lists, so the whole population is included in the study. The routinely collected data on antidiabetic drug utilization used in the study also came from all pharmacies (in-hospital and out-of-hospital) in Croatia, allowing a complete picture of antidiabetic drug utilization. Furthermore, only PHC doctors (family doctors and paediatricians) in Croatia are responsible for issuing drug prescriptions, including antidiabetics, which are reimbursed by the Croatian Health Insurance Fund (CHIF).

### 2.2. Morbidity Data

Morbidity data were obtained from the Croatian Health Service Yearbooks, which are publicly available at the website of the CNIPH [2]. Morbidity data from the yearbooks are based on the patients' records from all PHC practices around Croatia and since 2008 on patients' electronic records. The registration is based on the instructions for data registration and collection using WHO recommendation. According to those instructions, the first visit of a patient suffering from a chronic condition (such as DM) in a calendar year is registered as a morbidity case only once, independent of how many times he/she visited PHC [12].

In Croatia, the International Classification of Diseases version X (ICD-X) is used to register morbidity. Endocrine diseases are registered under E diagnoses. However, not all E diagnoses are present within the yearbooks. Endocrine diseases are grouped into four broad categories: thyroid disease (E00–E07), diabetes mellitus (E10–E14), obesity (E65-E66), and other endocrine, nutritional, and metabolic diseases. Because of those broad diagnostic categories, it is not possible to obtain separate data on Type 1 and Type 2; therefore, we will be only using the term DM. Furthermore, morbidity data are presented according to age group: 0–6 years, 7–19 years, 20–64 years, and over 65 years. All data were collected exactly in the way they were presented in the yearbooks over a 10-year period from 2005 to 2014. We calculated in percentages the ratio of E diagnoses in total morbidity, the ratio of DM diagnoses (E10–E14) within total E diagnoses, and the ratio of DM in relation to age groups. Rates of increases and decreases during the 10-year period were also calculated. Because the registration is based on one principle diagnosis per patient per year and because the entire Croatian population is included, it is possible to estimate the incidence and prevalence of DM in Croatia from this data.

### 2.3. Drug Utilization Data

Drug utilization data were obtained from yearly reports that are publicly available on the website of the Croatian Agency for Medicinal Products and Medical Devices (HALMED) [13]. The data obtained from these reports are based on data from all pharmacies, in-hospital and out-of-hospital, in Croatia. In the reports, utilization is presented according to both ATC/DDD methodology and financial indicators. The pharmaceutical utilization is presented in DDD per 1,000 inhabitants per day (DDD/TID) and spending is shown in the Croatian currency, kunas (December 2005 exchange rate: 1 EUR = 7.4 kunas, December 2014 exchange rate: 1 EUR = 7.7 kunas).

The following data were collected: total drug utilization and the utilization of antidiabetic drugs, including oral drugs and insulin listed under label A10 based on the ATC classification, for the period 2005–2014. We calculate in percentage the ratios of antidiabetic utilization within total drug utilization and the utilization of individual antidiabetics, for both, pharmaceutical usage (DDD/TID) and spending (in kunas). The ratios of increased/decreased antidiabetic utilization during the 10-year period were also calculated. According to the reports, only 2.98%–3.70% of drug utilization originated from in-hospital pharmacies [13]; therefore, the collected data were mainly related to antidiabetic drugs prescribed by PHC doctors.

The epidemiological descriptive observational method, free from artificial manipulation of the study data, was used in the study [14]. The collected data were analysed using Microsoft Excel package. The results are presented in absolute numbers, frequency (in %), and graphically, the trends are displayed as line charts.

## 3. Results

The results are presented in two parts: morbidity trends and antidiabetic drug utilization trends.

### 3.1. Diabetes Mellitus Morbidity Trends

**Figure 1:**
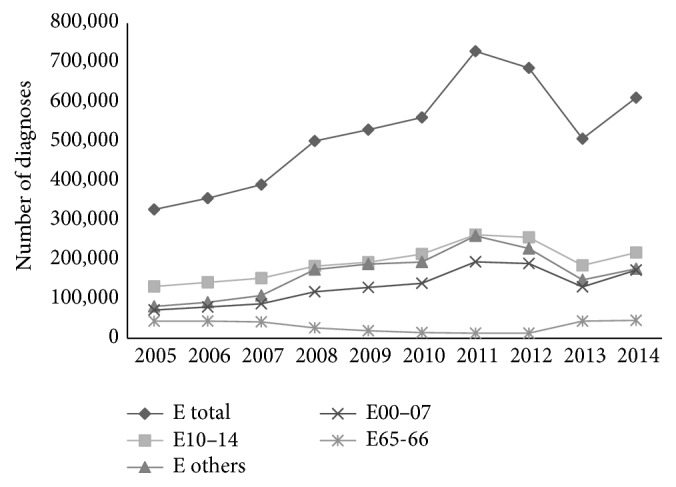
Trends in the number of endocrine diagnoses (E total; E00–E07, thyroid gland disorders; E10–E14, diabetes mellitus; E65-E66, obesity; E others, other endocrine, nutritional, and metabolic disorders) registered in primary health care (family practice and paediatrics) in Croatia, 2005–2014.

During the 10-year observational period, the total morbidity registered in Croatian PHC increased by 33.3%. The percentage of all E diagnoses among total morbidity also increased, from 4.2% to 5.9%. The total number of registered E diagnoses increased by 129% from 323,613 in 2005 to 742,195 in 2011, when it was the highest, and then it decreased to 617,176 by 2014. The highest increase of all of the E diagnoses was in the age group over 65 years (87.3%). Among the E diagnoses, the most frequent was DM (E10–E14), with a decreased share from 40.1% in 2005 to 35.5% in 2014. This was followed by the other endocrine, metabolic, and thyroid diseases. The lowest share belonged to obesity, which in some years was only 2% (2012). All of the diagnostic categories showed increases during the observation period, with thyroid gland disorders increasing by 140% and other endocrine and metabolic disorders by 118%. Only obesity decreased, from 43,296 registered diagnoses in 2005 to 12,657 in 2011, and then steadily increased to 45,063 in 2014 (Figure 1).

**Figure 2:**
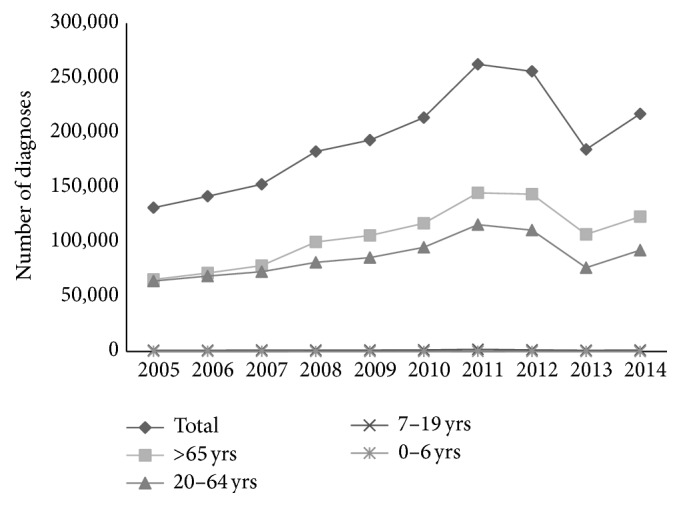
Trends in diabetes mellitus diagnoses registered in Croatian primary health care, family practice, and paediatrics, according to patient age, 2005–2014 (lines for the ages 0–6 and 7–19 years are overlapping).

During the observation period, the number of DM diagnoses registered in Croatian PHC increased by 65.6%, from 131,154 diagnoses in 2005 to 217,170 in 2014. The diagnoses were mostly related to the adult populations and the larger portion (50%–57%) belonged to patients over 65 years. In the age groups of 0–6 and 7–19 years, the shares decreased from 8.4% in 2005 to 5.9% in 2014. The increase in 86,143 diagnoses over the 10-year period included approximately 8,614 newly diagnosed/registered DM patients per year in Croatian PHC (Figure 2). According to this figure, the observed incidence of DM in Croatia is around 20 patients per 1,000 inhabitants per year. The observed prevalence in the adult population (2011 Census: 3,388,284 individuals older than 20 years) increased from 3.9% to 6.4% during the 10-year period. Furthermore, the increase was not equally distributed among the age groups, with the highest being the group over age 65 (87.3%), followed by group 20–64 years (43.7%). The number of DM diagnoses in the age groups of 0–6 and 7–19 years was stable (in Figure 2 presented as overlapping lines).

### 3.2. Antidiabetic Drug Utilization

**Figure 3:**
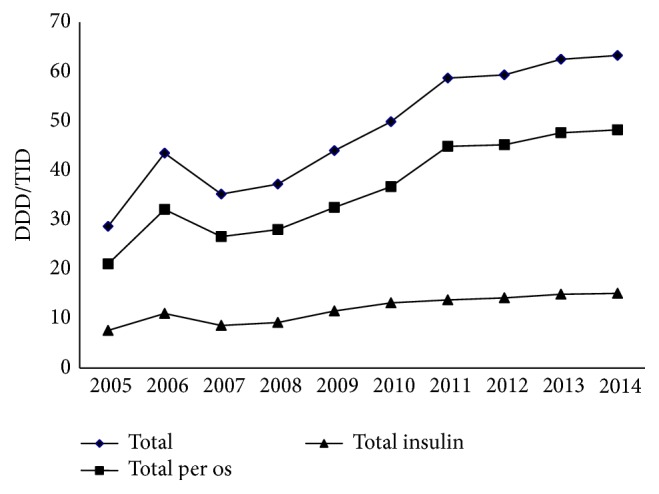
Trends in pharmaceutical consumption of antidiabetics (oral drugs and insulin) in DDD/TID in Croatia, 2005–2014.

From 2005 to 2014, pharmaceutical utilization of all antidiabetics increased by 120%, from 28.7% to 63.3% DDD/TID. It occupied between 4.3% and 6.5% of total drug consumption in Croatia. The consumption of oral antidiabetics increased by 129% (from 21.07 DDD/TID in 2007 to 48.19 in 2014) and consumption of insulin increased by 99% (from 7.6 to 15.1 DDD/TID in 2014). More than 75% of total consumption was of oral antidiabetics and the shares were stable from 2007 to 2014. During the final four years, the increase was not as sharp (Figure 3).

**Figure 4:**
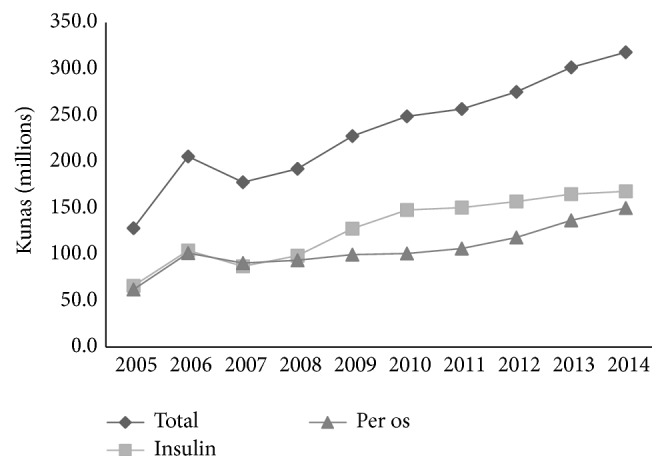
Trends in total spending (in millions of kunas) on antidiabetic drugs in Croatia, 2005–2014 (December 2005 exchange rate: 1 EUR = 7.4 kunas, December 2014 exchange rate: 1 EUR = 7.7 kunas).

Spending on all antidiabetics increased even more (147%), from 128.3 million kunas in 2005 to 316.8 million kunas in 2014, which was 3.4%–5.8% of total spending on drugs in Croatia. Spending on insulin was more than that for oral medications, with shares varying from 50% to 60%. Furthermore, spending on insulin increased even more, by 153%, from 66.1 million kunas in 2005 to 166.9 million kunas in 2014. Oral usage increased by 141%, from 62.2 million kunas in 2005 to 149.9 in 2014. The highest increase in oral drugs was during the final four years (Figure 4).

**Figure 5:**
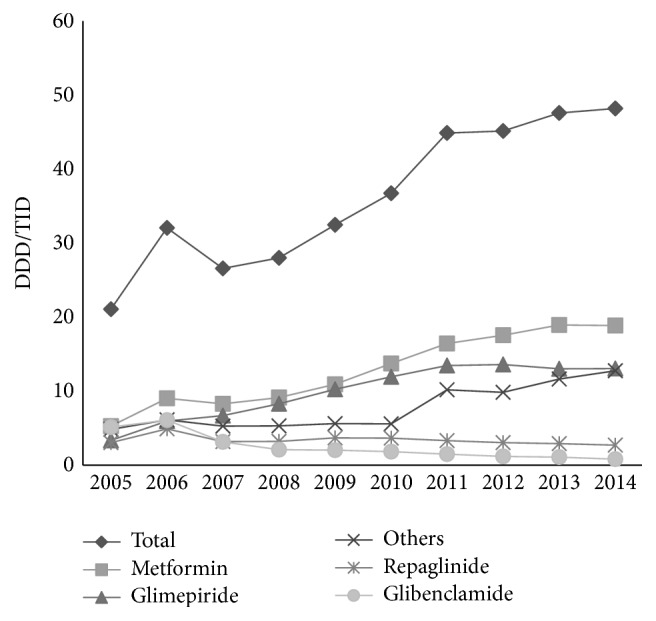
Trends in pharmaceutical utilization (in DDD/TID) of oral antidiabetic drugs in Croatia, 2005–2014.

Among oral antidiabetic drugs, metformin was the most widely used, comprising between 25% and 39.8% (2013) of total antidiabetic drug utilization, with a constant increase during the follow-up period, especially after 2009, from 10.9 DDD/TID in 2009 to 18.9 DDD/TID in 2014 for a total increase of 73.4%. Glimepiride was the second-ranked drug, with an even greater increase than metformin, from 3.4 DDD/TID in 2005 to 13.1 DDD/TID in 2014, for a total increase of 285%. The utilization trends for repaglinide were stable, while the use of glibenclamide continuously decreased (84.4%) from 5.2 DDD/TID in 2005 to 0.81 DDD/TID in 2014. Other antidiabetic drugs, such as dipeptidyl-peptidase 4 (DPP-4) inhibitors, alpha glucosidase inhibitors, and thiazolidinediones, were less utilized but with a constant increase (237.5%) from 0.8 DDD/TID in 2005 to 2.7 in 2014. Among these, the largest increase was with DPP-4, introduced in Croatia in 2009. By 2014, its usage had increased 890% from 0.02 to 1.8 DDD/TID (Figure 5).

**Figure 6:**
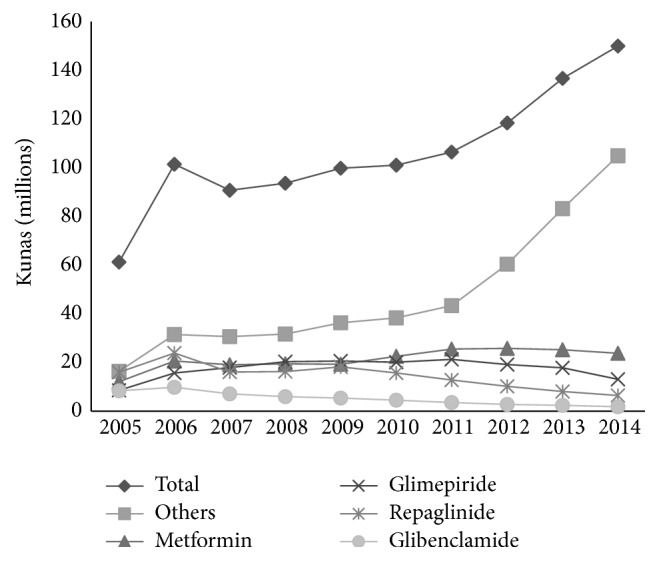
Trends in spending (in millions of kunas) for oral antidiabetic drugs in Croatia, 2005–2014.

The total spending on all oral antidiabetics followed the trend of pharmaceutical utilization, sharply increasing from 2005 to 2006, after which some antidiabetics increased and others decreased. The highest increase (104%) was observed in the drugs labelled “others,” mostly newly introduced DPP-4, and then metformin (96%) and glimepiride (51%). Again, the highest decreases were observed in glibenclamide (78.7%) and repaglinide (59.7%) (Figure 6).

**Figure 7:**
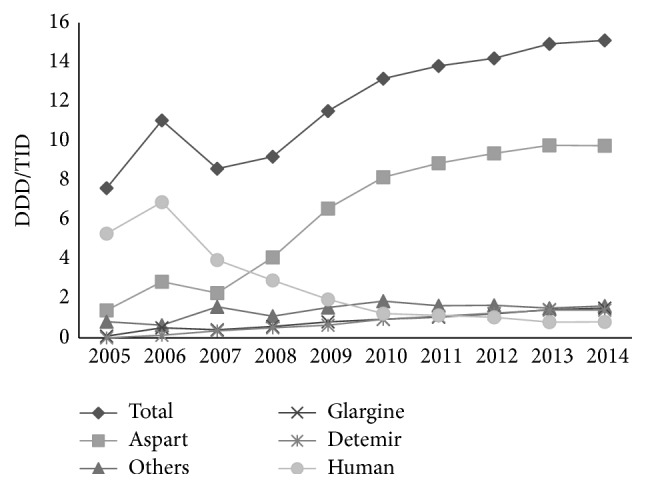
Trends in pharmaceutical utilization of insulin in DDD/TID in Croatia, 2005–2014.

Among all different kinds of insulin, the highest increase in pharmaceutical utilization was observed in aspart (600%) from 1.4 DDD/TID in 2005 to 9.8 DDD/TID in 2014, accounting for approximately 65% of all kinds of insulin in 2014. Trends in the utilization of human insulin suddenly increased in 2005–2006 and then constantly decreased by 88% until the end of the observation period. In comparison to others, the utilization of glargine and detemir was relatively small, but with the greatest increases. Glargine increased by 1566% from 0.09 DDD/TID in 2005 to 1.5 DDD/TID in 2014 and detemir increased by 907%, from 0.14 DDD/TID in 2006 to 1.41 DDD/TID in 2014 (Figure 7).

**Figure 8:**
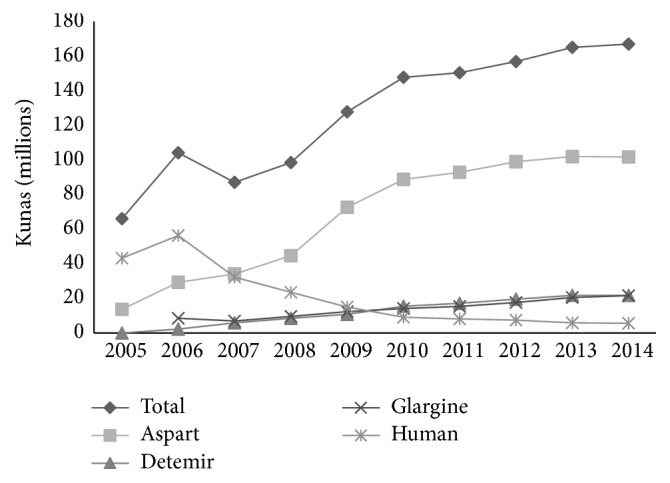
Trends in spending (in millions of kunas) on insulin in Croatia, 2005–2014.

Following the pharmaceutical usage trend, the total spending on insulin rapidly increased as well, especially after 2008. The increase was from 66.1 million kunas in 2005 to 166.9 million kunas in 2014, or 152%. As with pharmaceutical utilization, the highest increase in spending was for aspart insulin, growing from 20.8% to 60.9% of total insulin costs with an increase from 13.8 million kunas in 2005 to 101.8 million kunas in 2014. However, the increase in spending was higher than the increase in pharmaceutical utilization (600% for pharmaceuticals and 638% for spending). The increased trends were also observed for glargine and detemir insulin. For glargine, the increase was from 1.6 million kunas in 2005 to 21.7 million kunas in 2014, or 1256%. For detemir, the increase was from 5.8 million kunas in 2007 to 21.8 million kunas in 2014, or 276%. The trend for human insulin showed a sudden increase from 43.2 million kunas in 2005 to 56.3 million kunas in 2006. After that time, the trend constantly decreased to 5.7 mil kunas in 2014, for a total decrease of 87%. In 2014, total spending on insulin was 166.9 million kunas, with aspart accounting for 61%, human insulin for 3%, and glargine and detemir together for 26% (Figure 8).

## 4. Discussion

### 4.1. Main Findings and Comparisons with Other Studies

The obtained results indicate that the total morbidity in Croatian PHC increased by 33.3% during the 10-year observational period of 2005–2014. The total number of E diagnoses increased even more (129%), and the increase in the number of DM diagnosis was 65.6%. The largest portion of DM diagnoses belonged to patients over the age of 65 years (50%–57%), and the increase was the highest in this age group. According to the results, the average observed incidence of DM was 20 patients per 1,000 inhabitants per year and the estimated prevalence in the Croatian adult population increased from 3.9% to 6.4% during the observed period. Although there is no real data on the prevalence of DM in Croatia, this is still lower than in other Croatian studies based on limited data sources; in the 18–65 age group, DM prevalence was estimated to be 6.1% [15] and in all adults it was estimated to be 8.9% [16], while in the elderly population it was 15%–20% [17]. Based on the present results, it can be said that a high probability of increased trends is expected in the future, with approximately 8,614 newly diagnosed/registered DM patients per year in Croatian PHC. It is obvious that the prevalence of DM will double in the next 10 years and the burden of DM will increase, possibly more than for other chronic diseases.

The obtained results are only partly comparable to those in the literature because of different research methodology. In 2010, according to the International Diabetes Federation (IDF), the global prevalence of DM was 6.6%, and in European regions including Russia, DM prevalence is estimated to be 8.5% in the adult population [5]. Looking at the Southeast European Region Croatia belongs to, Turkey has the highest prevalence (14.8%), followed by Montenegro (10.1%), Macedonia (10.0%), Serbia (9.9%), and Bosnia and Herzegovina (9.7%). According to the WHO and the IDF, the incidence of DM is increasing all over the world, particularly in low- and middle-income countries. The socially disadvantaged in any country are the most vulnerable to this disease [1, 5]. Croatia is a middle-income country where the number of socially disadvantaged people is increasing, particularly during the current economic crisis.

Age is obviously an important risk factor for type 2 diabetes. We found that 50%–60% of DM diagnoses registered in PHC belonged to people aged over 65 years, while the share of this group in the total Croatian population in 2014 is 17.7%. In the European region, 37% of the population is over 50 years old, and the most prominent factor in the raising trend of DM is attributed to the aging of populations in Germany, Spain, Italy, France, and the UK [18, 19]. It is also obvious that obesity is an important risk factor [19, 20]. It is surprising that the number of patients with obesity constantly decreased in our study. This could be more a matter of “forgotten” diagnoses in Croatian PHC than of reality, because several studies have demonstrated increases in overweight and obesity in the Croatian population [21, 22].

The rising trend of DM prevalence could also be related to the clinical guidelines, which have constantly lowered the diagnostic level for blood glucose. According to the 1985 WHO criteria, DM was diagnosed by a fasting blood glucose (FBG) of >7.8 mmol/L [23]. In the American Diabetes Association (ADA) criteria from 1997, the diagnostic level for fasting plasma glucose (FPG) was set at >7.0 mmol/L [24], which was subsequently adopted by the WHO and by many nations, including Croatia [25]. The Croatian specificity is a mismatch of the diagnostic criteria (FBG > 7.0 mmol/L) and the therapeutic target level of blood glucose (FBG > 6.6 mmol/L) stated in the guidelines, which is often discussed among Croatian family doctors [25].

The situation with the diagnostic criteria for DM is even more complicated with regard to impaired fasting glycaemia and the diagnostic category of prediabetes [26, 27]. This phenomenon is often called overdiagnosis and overtreatment, as it unnecessarily labels a large number of people as ill, with no proven benefits [28–31]. According to small-scale research, the prevalence of impaired fasting glycaemia (by the WHO criteria) in Croatia is 11.3% [15]. We are not sure about the exact number of DM patients in this study to whom the diagnosis was established based on impaired fasting glycaemia and was labelled prediabetes. Furthermore, in Croatia, there is a lack of data on the number of reports of adverse reactions to statins, which are widely used, especially in diabetic patients [32–34]. The increase in HbA1c testing may also contribute to the rising DM prevalence, as was found in the USA [35]. During the last few years, HbA1c testing was introduced in Croatian PHC as a part of quality-improvement reimbursement schema, which could have some implications for the obtained results.

Increasing DM morbidity is followed by an even higher increase in antidiabetic drug utilization (morbidity increase, 65.6%; drug utilization, 120%). Metformin was the first-ranking oral drug, with a constant increase during the follow-up period. The second-most used drug, glimepiride, increased even more, while glibenclamide usage constantly decreased. High increase was observed in the group named “others,” mostly containing newly introduced DPP-4 inhibitors. Total utilization of insulin increased even more, mostly aspart and newly introduced glargine and detemir, while human insulin usage sharply decreased. Similar to the pharmaceutical trends, spending on antidiabetic drugs also increased because of the cost of insulin, mostly aspart, glargine, and detemir. However, the spending was not completely in line with pharmaceutical usage; spending on newly introduced drugs was much higher in comparison to their pharmaceutical consumption.

The increased metformin utilization was expected because this is in line with the Croatian guidelines of 2011 that suggested metformin as the first choice, similar to those stated at the ADA, EASD, National Institute for Health and Care Excellence, and other national guidelines [25, 36–40]. Comparable to our study, utilization of metformin has increased in all European countries [41, 42].

However, the finding in this study of the utilization of glimepiride as the second oral antidiabetic was somewhat unexpected, even more so if we consider a decrease in glibenclamide utilization. Sulfonylureas are usually added to metformin in many countries [39]. However, at this time there is no recommendation, or a consensus, on what the second-line drugs should be [43]. The reason for the frequent utilization of glimepiride in Croatia might be found in the Croatian, German, and NICE guidelines, which state that the combination of metformin with glibenclamide is associated with possibly unfavourable mortality data due to hypoglycaemia [38, 39]. There are several studies questioning the advantages and disadvantages of different sulfonilureas [44, 45]. However, the newer studies and meta-analyses have shown that there is insufficient evidence that the combination of metformin and different sulfonilureas is connected with all-cause and specific cardiovascular mortality [46, 47]. There is also no evidence of significant differences in side effects, including hypoglycaemic effects [39]. We are also not sure if pharmaceutical marketing might have some influence on the utilization of glimepiride as the first choice among sulfonilureas in Croatia. This phenomenon should be clarified in future research, even more so if we consider the higher price of glimepiride versus glibenclamide and the limited financial resources within the Croatian healcare system.

A similar and somewhat unexpected situation was found in relation to the consumption of insulin. While the percentage of Croatian DM patients on insulin in our study (23%) was similar to the UK (25%) [41], the US (27.1%) [48], and Portugal (20%) [42] and was lower than in Sweden and Norway (both around 50%) [42], the structures of insulin utilization are different. Contrary to other countries, the most often used one with the highest increase was aspart, while human insulin usage steadily decreased. This may relate to the Croatian guidelines, in which insulin analogues are given primacy [25]. According to the literature, despite some unimportant differences, human insulin are just as effective as the insulin analogues [49–52]. Based on this evidence, according to the NICE and many other guidelines, human insulin is recommended as the first-line insulin for treatment of type 2 DM and also because of the costs [38, 53]. In our study, the majority of spending was for aspart, increasing from 20.8% to 60.9% of total insulin costs during the observation period.

### 4.2. Strengths and Weaknesses

The strength of this study comes from its 10-year follow-up period, a timeline that is long enough to allow the determination of existing trends and to forecast future trends regarding the prevalence of DM and antidiabetic drug utilization in Croatia. The 10-year observational period was chosen because that is long enough to ensure that every DM patient on PHC doctors' lists could be seen and registered. According to the literature, more than 95% of patients are seen by their PHC doctors within three consecutive years. In a 10-year period, almost no DM patients would be missed [54]. Furthermore, the data come from the official national sources used for health-services planning in Croatia as well as for international comparisons. At the same time, the nature of the data was also a study limitation due to allowing the determination only of trends. Deeper insights, especially in terms of causal relationships in morbidity and antidiabetic drug utilization trends, are not possible in this type of research. The computerization of PHC and better registration, which started in 2005 and has been obligatory since 2008, might also have some influence on the increased number of DM diagnoses. In addition, the observed morbidity trends should not be mismatched with morbidity in theoretical meaning. As they mainly represent the reasons for patients visiting PHC, the trends in incidence and prevalence of DM are only estimations. Using routinely collected data does not allow us to make distinction between Type 1 and Type 2 DM, as well as between deferent age groups, which are also the study limitations. There is also some inconsistency with the morbidity data, for example, the sharp increase in the number of DM diagnoses in 2011 followed by decrease at the expected level, which could be attributed to the use of new computer software at that time. This should be taken into account in future data collections.

### 4.3. Implications for Practice

Beyond the limitations, the results of this study should be taken into consideration, firstly by PHC doctors, who should think about the scientific evidence and limited financial resources when working with diabetic patients. It will be also useful to think about introducing nonpharmacological measures for dealing with DM [55, 56]. It is even more important that, according the CroDiab 2012 data [57], only 26.9% of DM patients reached the targeted level of HbA1c, despite the increase in antidiabetic drugs utilization. It might be also worthy to invest time and professional energy in the revision of the Croatian DM guidelines to bring them in line with the new scientific evidence and for exclusive use in PHC. Furthermore, the results are also important for diabetologists because of their specific roles within the Croatian health care system. Large numbers of DM patients are under the care of diabetologists in Croatia; until recently, family doctors and paediatricians were not even allowed to start insulin therapy before sending a patient for a diabetologist consultation, and insulin analogues could be prescribed and reimbursed by the CHIF only upon a dialectologist's recommendation. The obtained results could serve policy makers as the basis for health service planning, including human resources planning and defining boundaries between PHC and secondary care, but also to guide future trends in continuing professional development of PHC doctors. The results of this study also challenge future researchers to obtain answers on real DM and prediabetes morbidity in Croatia and to investigate the deeper causal relationships in antidiabetic drug utilization trends.

## 5. Conclusions

Routinely collected data show that the number of registered diagnoses of diabetes mellitus in Croatia is greatly increasing. It is obvious that this trend will continue in the future. The utilization of antidiabetics is also increasing, with some discrepancies between pharmaceutical usages and spending, pointing once again to the importance of the cost of drugs. As expected, metformin is used as the first-line choice, but glimepiride was second, giving it an importance that is not evidence-based or seen in other countries and guidelines. There is a similar situation with insulin, giving primacy to analogues in comparison to human insulin.
